# Applications of cell penetrating peptide-based drug delivery system in immunotherapy

**DOI:** 10.3389/fimmu.2025.1540192

**Published:** 2025-01-22

**Authors:** Jing-Jing Du, Ru-Yan Zhang, Shangchi Jiang, Shanshan Xiao, Yiting Liu, Yongheng Niu, Wen-Xiang Zhao, Dongyuan Wang, XianShi Ma

**Affiliations:** ^1^ Hubei Key Laboratory of Kidney Disease Pathogenesis and Intervention, College of Medicine, Hubei Polytechnic University, Huangshi, China; ^2^ Department of Pharmacy, Union Hospital, Tongji Medical College, Huazhong University of Science and Technology, Wuhan, China; ^3^ Hubei Province Clinical Research Center for Precision Medicine for Critical Illness, Wuhan, China; ^4^ Department of Hepatobiliary Surgery, Yangxin County People’s Hospital, Huangshi, China

**Keywords:** cell penetration peptide, covalent conjugation, non-covalent delivery, cancer immunotherapy, clinical application

## Abstract

Cell penetrating peptides (CPPs) are usually positive charged peptides and have good cell membrane permeability. Meanwhile, CPPs are facile to synthesize, and can be functionalized to satisfy different demands, such as cyclization, incorporating unnatural amino acids, and lipid conjugation. These properties have made them as efficient drug-delivery tools to deliver therapeutic molecules to cells and tissues in a nontoxic manner, including small molecules, DNA, siRNA, therapeutic proteins and other various nanoparticles. However, the poor serum stability and low tumor targeting ability also hindered their broad application. Besides, inappropriate chemical modification can lead to membrane disruption and nonspecific toxicity. In this paper, we first reviewed recent advances in the CPP applications for cancer therapy via covalent or non-covalent manners. We carefully analyzed the advantages and disadvantages of each CPP modifications for drug delivery. Then, we concluded the recent progress of their clinical trials for different diseases. Finally, we discussed the challenges and opportunities CPPs met to translate into clinical applications. This review presented a new insight into CPPs for drug delivery, which could provide advice on the design of clinically effective systemic delivery systems using CPPs.

## 1 Introduction

Due to the hydrophobic nature of the therapeutic molecules and their low bioavailability *in vivo*, many promising therapeutic drugs, especially those targeting intracellular therapeutic molecules, face challenges in fully realizing their therapeutic efficacy (1, 2). Enabling these molecules to cross the cell membrane so that they can have a therapeutic effect is a huge challenge.

In the late 1980s, while studying the human immunodeficiency virus (HIV), Green’s group discovered that the protein-transduction domains (PTD) of the transcription-activating protein Tat was able to penetrate cell membranes *in vitro*, and further studies revealed that the amino acid sequence corresponding to residues 48-60 of Tat (RKKRRQRRR) played a key role in cellular uptake (3–5). Subsequently, peptides found to penetrate cell membranes were commonly defined as cell-penetrating peptides (CPPs). CPPs have short sequences (typically less than 40 amino acids) and are usually cationic peptides, such as the two earliest identified CPPs (Tat and the Antp) (3, 4, 6), which have been shown to be capable of transporting a large number of cargoes into the intracellular environment. In addition to naturally derived sequences, many chimeric and synthetic peptides have been designed for drug delivery. Based on the type and arrangement of amino acids, CPPs are categorized as cationic, anionic, amphipathic and hydrophobic peptides (7). CPPs are usually structurally composed of 5-30 amino acids, which are divided into natural peptides and pure synthetic peptides depending on their source. They are able to pass through cells and tissues through various mechanisms, for example, cationic CPPs interact electrostatically with negatively charged carboxyl and phosphate groups on the cell membrane, whereas hydrophobic peptides may be transported by interacting with hydrophobic regions of the cell membrane (8–10). To date, CPPs have been widely used to deliver cargoes such as small molecule drugs, proteins, nucleic acids, etc. However, the specific mechanism of cellular uptake of CPPs is not yet fully understood, which may depend on the type and concentration of cargoes and the temperature (11). In conclusion, the discovery of CPPs provides an opportunity to deliver molecules with intracellular therapeutic activity.

This review will focus on the study of CPPs in facilitating intracellular delivery of a variety of cargo molecules, including the strategies of covalent conjugation and self-assembly. We will also present recent advances in the use of CPPs for clinical trials targeting different diseases and summarize the barriers to the translation of CPPs into clinical drugs.

## 2 Covalent conjugation of CPPs

**Figure 1:**
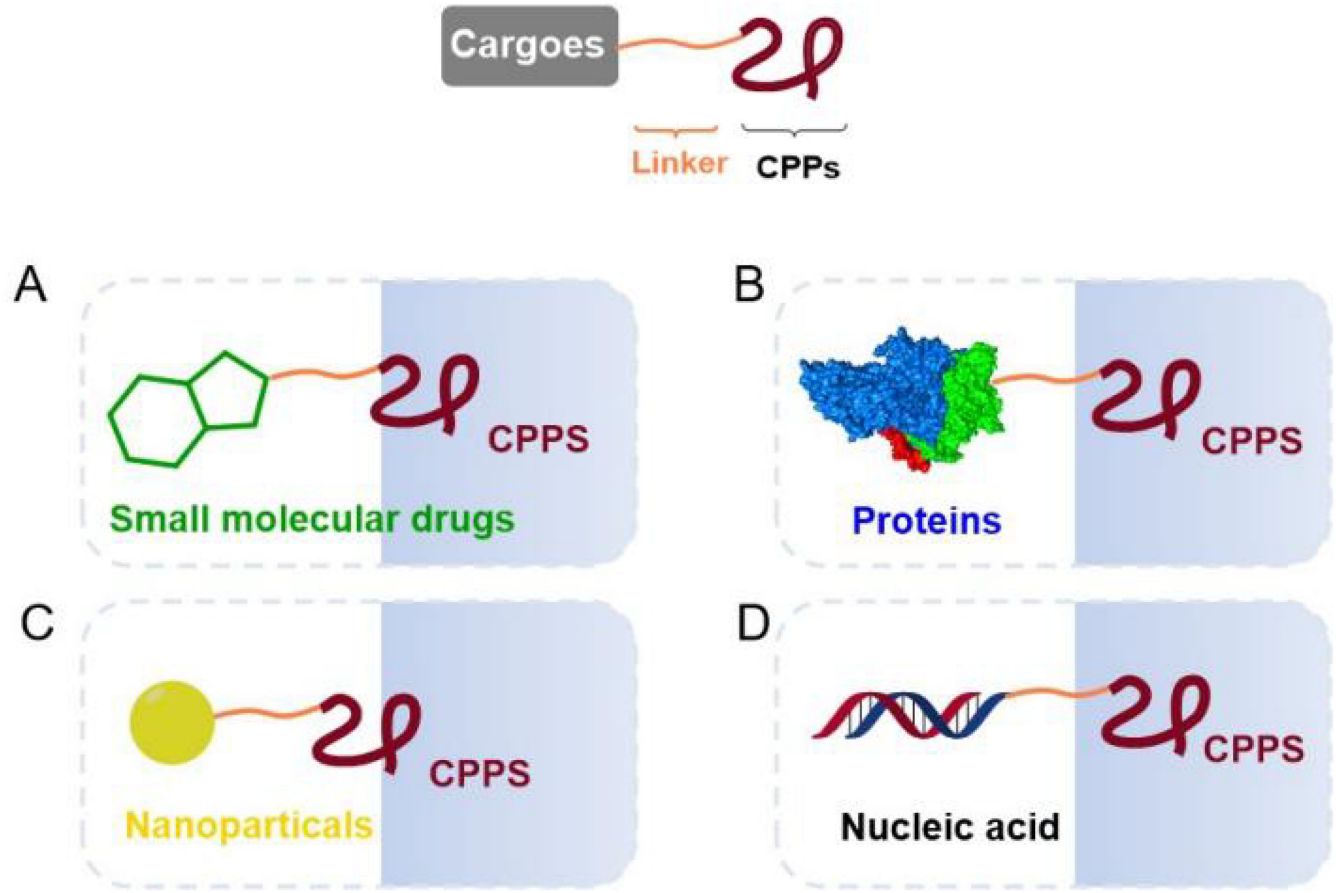
Covalent conjugation strategies based on CPPs and biomolecules in drug delivery system (12–19).

As a class of peptides with special functions, CPPs are able to carry a variety of biomolecules across the cell membrane (**Figure 1**), including small molecule drugs (12–14), proteins (15, 16), nanomaterials (17), and nucleic acids (13, 18, 19), which provide novel delivery strategies for molecules that are impermeable to cells. In recent years, covalent coupling of vehicles to therapeutic molecules has been one of the hot topics in drug delivery system researches, such as antibody drug conjugates and peptide drug conjugates (20, 21). ADC is formed by connecting monoclonal antibodies and potent cytotoxic payloads through chemical linkers, while PDC is formed by combining cargo peptides and cytotoxic payloads through linkers, both of which enhance their tumor selectivity and permeability. Covalent coupling involves the formation of a chemical bond to maintain the integrity of the CPP and the cargo while forming a stable complex. This approach ensures that the cargo binds tightly to the CPP during circulation, which keeps its free from enzymatic degradation and increasing its half-life and bioavailability (1). In addition, covalent conjugation avoids non-specific distribution of cargoes, improves drug accumulation in the target cells and reduces off-target effects to a certain extent (22).

### 2.1 Covalent conjugation with small molecule drugs

Small molecule drugs have been widely used in the treatment of cancer, bacterial and viral infections (23–25). However, the half-life of small molecule drugs is too short and their poor membrane permeability restricts their ability to reach the treatment site effectively. Therefore, an excessive amount of small molecule drugs is usually required to achieve therapeutic effects. Additionally, some small molecule drug candidates exhibit off-target cytotoxicity. In recent years, more studies have focused on how to effectively combine peptides with anticancer drugs for targeted delivery (26).

The Hristova group has reported a cellular transmembrane peptide, called CL peptide, containing a helical motif (RLLRLLR) and a polyarginine tail (r7). The peptide-cargo coupling increased the transport of small-molecule cargoes by approximately 10-fold and efficiently released cargoes (27). In 2012, the Qi group developed a conjugate (ACPP-DOX) of activatable cell-penetrating peptide (ACPP) with the antitumor drug adriamycin (DOX) for tumor-targeted therapy. Cellular uptake of ACPP-DOX was enhanced upon enzyme-triggered activation, and the internalized DOX was effective in inhibiting the proliferation of HT-1080 cells (28). Singh et al. designed and synthesized an octreotide-oxaliplatin concatenate (pcp-oxal). This coupling rapidly delivers oxaliplatin into cells, thereby not only improving intracellular delivery efficiency, but also enhancing passive targeting *in vivo*. The internalized oxaliplatin effectively inhibited tumor growth and exhibited considerable antitumor activity, indicating that the conjugate demonstrates good efficacy*in vivo (* 29). Backlund et al. conjugated CPP with 10 peptides representing neoantigens, tumor virus antigens, and the tumor associated antigens in their study, and concluded that nine of the peptides exhibited enhanced T cell primers, demonstrating that conjugating CPP with antigens in vaccine administration could enhance therapeutic control of tumors (30). In 2024, Shi’s group developed a cell penetrating peptide induced chimeric conjugates (cp-PCCs) and used them to induce degradation of palmitoyltransferase DHHC3. This method disrupted the immunosuppressive function of PD-L1 by reducing its palmitoylation and membrane retention, thereby enhancing the immune response to tumors (31). In conclusion, delivery of small molecule drugs by covalently coupled CPPs is a promising research area that not only expands the possibilities of drug delivery but also brings new hope for the treatment of diseases.

### 2.2 Covalent conjugation with proteins

The lipophilic components of the plasma membrane can act as a barrier, preventing proteins from easily reaching intracellular targets. Peptide-based delivery systems offer various advantages such as low immunogenicity, a high safety profile, and controlled dosage of administration (32, 33). The “YGRKKRRQRRR” sequence from the HIV Tat protein, commonly referred to as the Tat peptide, has been widely utilized. In 1994, Fawell et al. chemically crosslinked Tat peptides to a variety of proteins, including β-galactosidase, horseradish peroxidase, and the structural domain III of Pseudomonas exotoxin A (PE). The Tat successfully translocated different types of proteins into the cell, indicating that Tat-mediated uptake might allow delivery of macromolecules previously thought to be impermeable to living cells (34). Since then, researchers have developed various covalent conjugates to CPPs.

CPP-protein fusions with recombinant DNA technology have been used for many cargo proteins, including enzymes (35, 36), antibodies (37, 38), and antigens (39–42). Berne’s group constructed recombinant fusion proteins by fusing five different CPPs to the antibody, respectively, and demonstrated that the CPP-antibody fusions significantly enhanced antibody penetration into cells. This study offers new insights for further exploration of therapeutic antibodies against intracellular targets (38). Jiang’s group genetically constructed a GFP-Tat fusion protein that incorporates sequencing enzyme-mediated protein cyclization and cell-penetrating peptide (CPP)-mediated intracellular delivery improved the efficiency and stability of intracellular protein delivery (43). Cyclized GFP-Tat (cGFP-Tat) highly retained the photophysical properties of the protein and significantly improved stability *in vitro* with better intracellular delivery efficiency and tumor retention *in vivo*.

With the advancement of click chemistry, azide-functionalized CPPs were chemically coupled to alkyne-functionalized proteins by copper-catalyzed azide-alkyne cycloaddition (CuAAC) in order to construct stable site-specific structures. Christian’s group utilized the azide-functionalized polyphosphorylated adenosine and alkyne-functionalized polyphosphorylated adenosine to obtain cyclic and linear conjugates (44). The resulting cyclic CPP-GFP coupling was efficiently internalized into living cells, whereas the linear CPP analogue failed to facilitate GFP transduction. Kulkarni’s group constructed a coupling consisting of a growth factor receptor-binding protein 7 (GRB7) inhibitor, fitc-labeled penetrant peptide (CPP), and nuclear localization signal (NLS). The resulting GRB7-CPP-NLS structure greatly enhanced cellular uptake and localization to the cytoplasm and nucleus of breast cancer cells (45).

The covalent conjugation of CPP with cargo peptides or proteins could be achieved through chemical means. This involves the use of specific linkers to conjugate disulfide and amine bonds, ensuring the inherent proximity of the CPP to its cargo, thereby promoting cargo release once the linker is internalized into the cell. Through this method, CPP could be used as a carrier for peptide and protein delivery, and applied to target cancer (46–49). Currently, protein/peptide therapy is mainly used for regulating diseases in the extracellular space. In 2023, Zhao et al. developed peptides with pH dependent membrane perturbation activity by replacing Arg/Lys residues in cationic CPP with histidine, which facilitated intracellular escape of chromosomes in the context of CPP. They found in their study that the fusion of trastuzumab hsLMWP BID with 16 residue peptide (hsLMWP) and pro apoptotic protein BID (BH3 interacting domain death agonist) had potent anti-tumor efficacy, and demonstrated minimal side effects (50).

### 2.3 Covalent conjugation with nanoparticles

Nanoparticles are materials with sizes between 1-1000 nm, including metals, polymers, vesicles (e.g., micelles and liposomes), and carbon-based materials (e.g., nanotubes, fullerenes, and nanodiamonds) (51). In recent decades, nanomaterials have received increasing attention in the medical field due to their large surface area and favorable properties for controlled absorption and release. The diversity of nanomaterial types influences the mechanisms and effectiveness of their conjugation with CPPs. Based on these distinct categories, nanomaterials can be classified into several groups, including inorganic nanomaterials (e.g., gold and silver) (52) magnetic nanoparticles (53), polymer-based nanoparticles (54), liposomes, and vesicular systems such as micelles. Owing to their unique physicochemical properties, these various classes of nanomaterials exhibit specific advantages and application potentials when conjugated with CPPs, thereby providing a range of solutions for nanomedicine.

Because of their superior internalization and transmembrane transport capabilities, CPPs are considered an effective carrier for moving NPs through cell membranes. In 1999, Weissleder et al. (55) made the first attempt to produce CPP sequence-derived particles, which showed a 100-fold higher rate of internalization of the conjugate in lymphocytes compared to unmodified particles. Since then, researchers have continuously sought to bind CPPs to NP surfaces using covalent coupling techniques. Among metal nanoparticles, gold nanoparticles (GNPs) currently attract the most attention in drug delivery systems (53, 56–59). GNPs are suitable for targeted delivery, bioimaging, and theranostics due to their reduced toxicity, ease of modification, and excellent biodistribution when conjugated with CPPs. CPP-conjugated GNPs have enhanced cellular internalization and are suitable for various biomedical applications as nano-conjugates.

Additionally, polymer nanoparticles are attractive options for the delivery of cargoes. Among various polymer nanoparticles (54, 60–63), poly(lactic-co-glycolic acid) (PLGA) NPs have proved to be remarkably successful in combating a wide range of conditions including infectious diseases and cancer. Researchers successfully attached CPP to the surface of NPs using different surface-modification chemistries such as avidin and DSPE-PEG. Compared with unmodified NPs, CPP-modified NPs greatly improved cellular internalization, offering a promising delivery option for NP applications (62, 64, 65).A PROTAC strategy utilizing a covalent nanobody (GlueBody), known as GlueTAC, has been proposed for the targeted degradation of membrane proteins. Zhang et al. developed GlueBody through a mass spectrometry-based screening platform and successfully constructed a GlueTAC chimera that is covalently linked to cell-penetrating peptides and lysosomal sorting sequences (66). This chimera effectively triggers the internalization and degradation of programmed death ligand 1 (PD-L1), thereby providing a novel approach for targeting and degrading cell surface proteins. Leveraging the tunable chemical and physical properties of nanoparticles, along with surface functionalization strategies, allows for enhanced cell specificity. The integration of cell-penetrating peptides (CPPs) with nanoparticles (NPs) shows significant potential for enhancing cellular uptake, facilitating targeted drug delivery, and advancing anti-cancer therapies (54). As targeted delivery systems undergo continuous refinement, advancements in the CPP-NPs domain are poised to further enhance the application of CPPs in cancer research.

### 2.4 Covalent conjugation with nucleic acids

Due to their high internalization efficiency, low cytotoxicity, and flexible design, CPPs are a promising strategy for delivering nucleic acid drugs, including genes, short oligonucleotides, and small interfering RNAs (67, 68).

As a promising gene therapy strategy, specific gene silencing by RNAi has required the delivery of RNA into the cytoplasm. However, since RNA is negatively charged, it is difficult to cross the cell membrane due to strong repulsion by the negatively charged plasma membrane (69, 70). CPPs are cationic peptides capable of delivering oligonucleotides, which makes them highly promising delivery vectors.

Jagrosse et al. reported a structure-function study of CPPs-RNA conjugation using a series of modified cyclic amphipathic cell-penetrating peptides (CAPs), which have been shown to effectively deliver RNA. The researchers examined the effects of different peptide sequences on siRNA binding efficiency, cellular delivery and knockdown efficiency, and endocytosis uptake mechanisms (71). The results demonstrate that the strong cationic character and the aromatic residues capable of participating in CH-π interactions make CAP sequences the most effective in interacting with siRNA. The cyclic cationic CAP has exhibited a high siRNA translocation efficiency, contributing to the efficient knockdown of siRNA targets. Most CAP-siRNA complexes achieved siRNA delivery by clathrin- and caveolin-mediated endocytosis (72).

## 3 Non-covalent conjugation strategies of CPPs in immunotherapy

**Figure 2:**
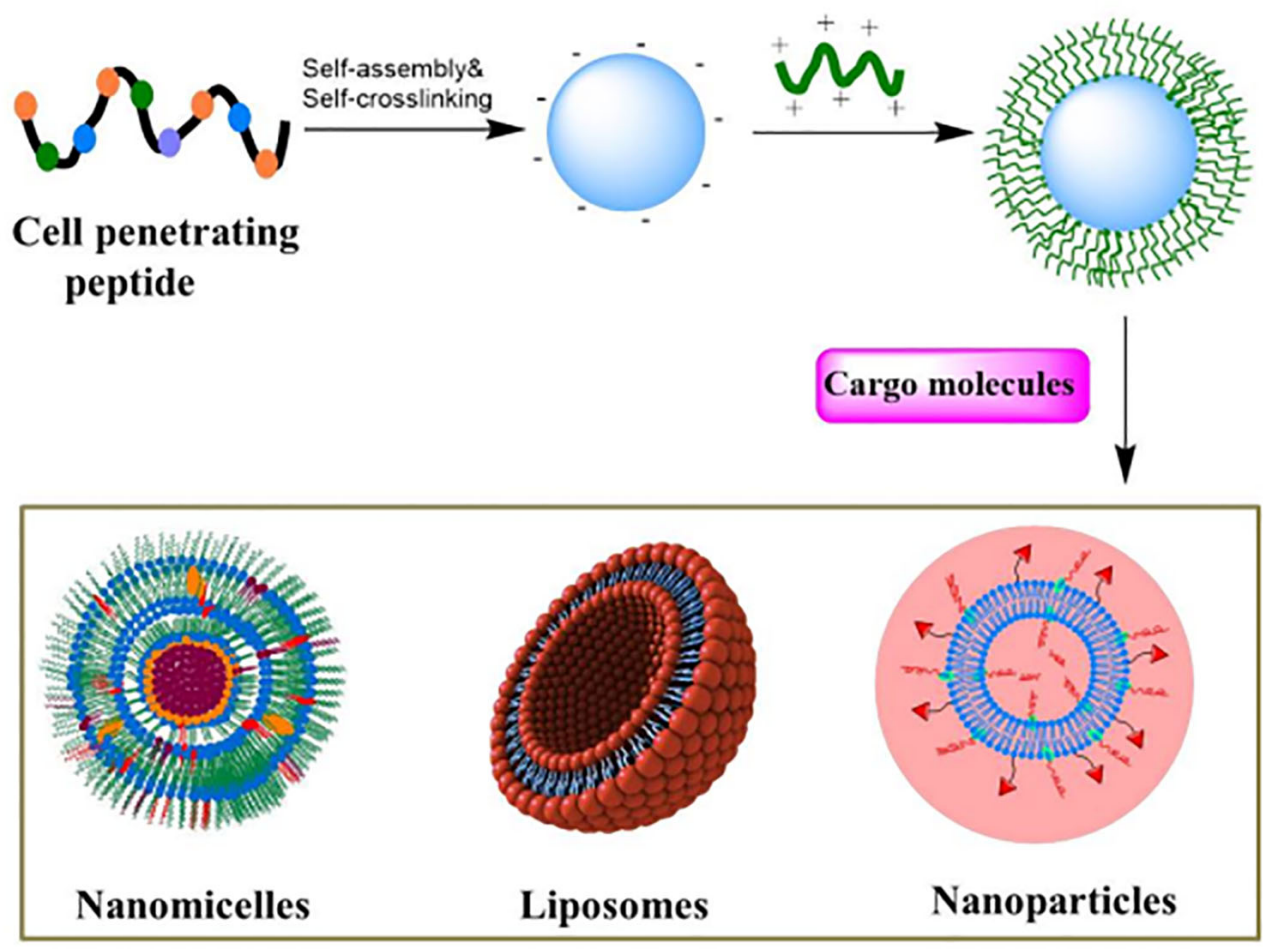
The main carrier system based on CPP functionalization is used for delivering therapeutic agents, which are loaded in their respective carrier systems.

CPPs not only easily penetrate cell membranes but also enter specific organelles, improving the accuracy of targeted therapy. Targeting specific organelles is critical when treating cancer, as targeted delivery of anticancer drugs to specific intracellular targets improves therapeutic efficacy and reduces drugs toxicity. Current research shows that CPPs, combined with nanomicelles, liposomes and nanoparticles loaded with anticancer drugs, enhance drug transport across the blood-brain barrier and improve targeted treatment of tumor cells, offering greater control over drug delivery (**Figure 2**) (73). Due to the inherent and easily modifiable properties of most CPPs, they are particularly suitable for CPPs assembly and drug delivery applications to achieve higher therapeutic efficacy (74–77). In conclusion, CPPs are promising tools for improving cellular uptake, and CPPs binding lipid nanoparticles loaded with anticancer drugs have promising applications in cancer therapy (78).

### 3.1 Self-assembling into micelles

Nanomicelles, with their outstanding biocompatibility, low toxicity, high drug carrying capacity, tiny size, and prolonged drug release time, and can be employed to reduce anticancer medication toxicity and avoid drug resistance (79). So far, nanosystems based on micelles for diagnosis and therapy have been extensively studied, and nano-drugs derived from micelles have emerged as a major treatment option for cancer. These micelles have a nanoscale structure, and their size (10-100 nm) allows them to penetrate deep into vascular tissues, such as tumors, by utilizing a porous vascular system. The effectiveness of CPP assembled micelles can be regulated by various external triggering factors, such as local arginine concentration, pH value, and temperature. Scientists have developed a series of strategies, including modified block copolymers coupled with cell penetrating peptides that better assemble into micelles, form stable complexes with siRNA, or load anticancer drugs and effectively delivering them to brain tissue via intranasal delivery (80).

In recent years, various studies on the combination of nanomicelles have been conducted, with the goal of providing new cancer therapy alternatives. Tat, a cell-penetrating peptide derived from HIV Tat (GRKKRRQRRRCG), and MPEG-PCL Tat are synthesized by Tat using disulfide bonds. They first self-assemble into micelles and then form stable complexes with siRNA, or can be loaded with anticancer drugs for effective delivery. Kanazawa et al. demonstrated the efficiency of drug/siRNA co-delivery and nasal brain delivery using MPEG-PCL-Tat nanomicelles in the treatment of glioblastoma and also exhibited its potential for treating brain and central nervous system-related diseases (81). In another work, Weinberger et al. designed drug molecules to self-assemble into spherical micelles, and then decorated soft nanospheres with an arginine-rich CPP (Tat) sequence on the membrane to restore the binding between CPP and lipid bilayers, improving the efficiency of CPP in delivering drugs *in vivo* and providing a more effective method for CPP-based cancer treatment (82).

To provide more targeted treatment for glioblastoma, efforts have been made to enhance the specificity of nanomicelle-related drugs for glioblastoma. For example, Zhu et al. proposed tandem nanomicelles co-functionalized with glioblastoma-targeting and cell-penetrating peptides, Angiopep-2 and Tat. Tandem nanomicelles with 20 mol% Angiopep-2 and 10 mol% Tat highly enhanced the specificity of anti-glioma therapy, improved survival rates, and had minimal side effects (83). Another study showed that, RRR-a-tocopheryl succinate-grafted-ϵ-polylysine conjugate (VES-g-ϵ-PLL) self-assembled ultra small micelles (NMs) served as delivery vehicles for chemotherapy, such as the hydrophobic model drug docetaxel (DTX). This study demonstrated that DTX micelles (DTX NMs) with a drug loading of 3.08% combined with ultrasound-targeted microbubble disruption (UTMD) could induce more significant apoptosis in C6 tumor cells, effectively overcoming the blood-brain barrier and treating glioblastoma (84).

### 3.2 Self-assembling into liposomes

The delivery of anti-tumor drugs from the systemic circulation to tumor sites is impeded by various physiological and biological barriers, including the blood-brain barrier (BBB) and the limited permeability of these drugs within tumors. To address this challenge, Pirhaghi et al. demonstrated experimentally that covalent coupling or modification of various peptides with liposomes loaded with anti-tumor agents can significantly enhance the capacity of these liposomes to traverse the blood-brain barrier (85). Given their relatively high cellular permeability, liposomes are extensively used to improve drug entry efficiency into cells (86, 87).

For instance, poly-L-arginine acts as a cell-penetrating peptide (CPP), significantly improving the endocytosis efficiency of the liposomes. Sharma et al. investigated a novel dual-ligand liposome carrier incorporating transferrin and poly-L-arginine to enhance the efficacy of drug delivery across the blood-brain barrier. Specifically, transferrin served as a targeting ligand, covalently conjugated to specific chemical moieties on the liposome surface, such as amide bonds or thiol groups, thus facilitating targeted delivery to the brain (88). Furthermore, Liu et al. developed a dual-mediated targeted liposome called transferrin-cell-penetrating peptide-electrostatically stabilized liposome (TF-CPP-SSL). This system integrated transferrin receptors (TF-R) and cell-penetrating peptides (CPP), promoting efficient drug delivery to gliomas. During this process, the researchers employed PEG modification to enhance the stability of the liposomes in circulation and optimized both the concentrations and modification densities of TF and CPP to ensure optimal cellular targeting and endocytosis efficiency. The findings indicated that TF-CPP-SSL could effectively traverse the blood-brain barrier (BBB), undergo endocytosis in C6 glioma cells, escape lysosomal degradation, and release drug components, such as doxorubicin into the cytoplasm for pharmacological action (89). In a subsequent study, Lakkadwala et al. developed a dual-functional liposome delivery system that used transferrin (Tf) to modify the surface of liposomes for enhanced receptor-mediated transport while concurrently introducing a cell-penetrating peptide (Pen) to improve cellular uptake efficiency. This formulation effectively encapsulated the chemotherapeutic agents doxorubicin (Dox) and erlotinib (Erlo), enabling precise traversal across the blood-brain barrier and targeted delivery to glioblastoma. Experimental results indicated that Tf-Pen liposomes significantly increased drug accumulation in brain tumors, with approximately 12-fold enhancement for Dox and approximately 3.3-fold for Erlo, while demonstrating favorable anti-tumor effects—over 90% tumor regression—and extending the median survival time of mice to 36 days (90, 91).

In further investigations, Shi et al. developed paclitaxel-loaded liposomes (PTX-TR-Lip) by combining TR peptide with pH-responsive and integrin αvβ3-specific carriers, effectively promoting penetration through the blood-brain barrier to target gliomas (92). Additionally, Li et al. constructed paclitaxel-loaded liposomes by incorporating the cell-penetrating peptide dNP2 along with pH-sensitive folic acid (FA), significantly enhancing cellular permeability and augmented the tumor-targeting efficacy of chemotherapeutic agents (93). Furthermore, Shi et al. developed a dual-functionalized thermosensitive lipid system (DOX@P1NS/TNC-FeLP) that integrates a glioma-specific cell-penetrating peptide (P1NS) with an anti-glioma antibody (TN-C), enabling precise traversal of the blood-brain barrier (94). Subsequently, Li et al. found that the co-utilization of transport protein (TP) peptides significantly enhanced the cellular uptake efficiency of liposomes. As an amphipathic cell-penetrating peptide, TP peptides facilitate paracellular uptake of liposomes via specific receptors and demonstrate high sensitivity to inhibitors targeting macrophage phagocytosis pathways. This finding establishes a foundation for the novel applications of TP peptides in cancer therapy (95).

### 3.3 Self-assembling into nanoparticles

CPPs, as a class of polycationic molecules, effectively facilitate the intracellular uptake of nanoscale cargo. The CPP-nanoparticle hybrid system represents an innovative approach in the fields of drug delivery and molecular biology This hybrid system integrates the cell-penetration capabilities of CPPs with the versatility of nanoparticles to enhance the delivery efficiency of therapeutic agents and genetic materials, effectively targeting tumor cells, reducing side effects, and improving therapeutic outcomes. To further explore the applications of CPPs, Moataz Dowaidar et al. conducted studies on their classification, absorption mechanisms, and hybrid carrier systems involving nanoparticles, highlighting the potential of CPPs for transporting siRNA and other cargo (96).

Researchers have extensively utilized nanoparticles modified with CPPs to deliver anticancer chemotherapeutic drugs to the brain for glioma treatment. For instance, Lakkadwala et al. developed a dual-functional liposome delivery system by combining CPPs with transferrin lipid nanoparticles while loading 5-fluorouracil (5-FU), successfully crossing the blood-brain barrier and significantly increasing 5-FU accumulation in tumor cells, along with its antitumor efficacy (97). Additionally, Kang et al. described a novel CPP characterized by an amino acid sequence comprising serine-isoleucine-tyrosine-valine (SIWV), which demonstrated significant homing ability toward glioblastoma brain tumors both *in vitro* and *in vivo*. They also investigated the potential combination of this CPP with porous silicon nanoparticles (psiNPs), which markedly enhanced selectivity and therapeutic efficacy in glioblastoma mouse models (98). Besides, tumor imaging is also a crucial step in the process of tumor treatment In 2021, Dai et al. explained that combining highly active aggregation-induced emission nanoparticles with PEG-polymers enhanced the biological activity of nanoparticles, which was more beneficial for tumor imaging and increased the accuracy of tumor diagnosis (99).

Furthermore, Silva et al. performed functionalization experiments on well-characterized nanolipid carriers (NLCs) using a straightforward and efficient adsorption method with three distinct peptide sequences. Zeta potential analysis confirmed successful peptide adsorption and indicated that various non-covalent interactions may be involved in this process. Computer simulations revealed a substantial interaction between CPP MAP and the NPY Y1 receptor, suggesting its potential significance in biological applications (100). Lastly, in 2023, Sugimoto et al. developed a highly functional KK-(EK)4 lipid and assessed its efficacy as a novel CPP-modified lipid for enhancing intracellular nanoparticle transport. They found that, compared to unmodified exosomes (EVs) and mRNA-encapsulated lipid nanoparticles (mRNA-LNPs), KK-(EK)4-lipid-modified carriers exhibited significantly improved cell-binding capacity and enhanced *in vitro* protein expression levels—further underscoring the promise of CPPs for intracellular delivery applications (101). Collectively, these studies underscore that the integration of CPPs with nanoparticles provides critical support for advances in modern medicine and biotechnology.

## 4 Application of CPPs in clinical trials

CPPs have demonstrated significant potential for clinical applications in oncology, particularly in cancer prevention and treatment, garnering increasing attention from researchers (102). These peptides not only exhibit a remarkable capacity to efficiently traverse cell membranes but also possess the ability to selectively target specific cellular organelles with large biomolecules, thereby greatly enhancing drug delivery efficiency while minimizing side effects. This advancement offers novel insights and directions for cancer prevention and treatment (103, 104). Some studies have shown that some CPPs have been used in clinical studies to inhibit tumor growth. For example, based on the evidence provided by clinical pharmacological research that there is no significant toxicity or immunogenicity, p28 has entered phase I clinical trials. In clinical practice, the combination of CPP DTS-108 and the antirectal cancer drug irinotecan significantly reduces gastrointestinal cytotoxicity compared to using irinotecan alone (105). Fifteen patients received intravenous injections of p28, which showed good tolerability and safety, indicating that p28 appears to have anti-tumor activity in advanced cancer patients (106). These findings underscore the extensive clinical applicability of CPPs in tumor management. Previous preclinical investigations indicate that CPP-based therapeutic strategies not only yield promising outcomes in oncology but also offer fresh perspectives on treating various other diseases. As clinical research progresses, it is anticipated that CPPs will assume an increasingly pivotal role in oncological treatments.

### 4.1 The clinical trial of activatable cell penetrating peptides

ACPPs are a novel class of *in vivo* targeted drugs, formed by a CPP that binds to a polyanion through a cleavable linker. Jiang et al. proposed the mechanism of ACPPs, where cleaving the linker to break down its structure, allowing the cationic peptide and its cargo to attach or enter the cell. Then, matrix metalloproteinases (MMPs) were used to cleave ACPP and combine with fluorescent groups for tumor imaging. This could concentrate molecules on cells and in areas adjacent to extracellular lytic activity within cells, ACPP became a new strategy for selectively delivering molecules to tumor cells (107). Subsequently, various studies were conducted on ACPP. For example, in 2009, Olson et al. demonstrated through their study of the structure and *in vivo* effects of ACPPs that ACPPs have the advantages of high resolution, enzyme specificity, and *in vivo* tumor imaging. Additionally, due to their elevated permeability, ACPPs can serve as an effective sensor for *in vivo* proteases. At the same time, they also showed that ACPP could target numerous xenograft tumor models from different cancer sites, including spontaneous breast cancer transgenic models (108). These studies indicate that ACPP has great potential in tumor research.

### 4.2 The clinical trial of therapeutic agent p28

p28 is an effective therapeutic agent that can serve as a tumor-targeting carrier molecule to preferentially penetrate cancer cells (109). It is highly water-soluble and stable, and no significant side effects or immunogenicity were observed in clinical treatment. The primary objective of the study through the Phase I clinical trial was to determine the level of no observed adverse effects (NOAEL) and maximum tolerated dose (MTD) of p28 in adult patients with advanced solid tumors. These patients had advanced tumors that did not respond to conventional treatments and were expected to survive for approximately six months in this setting. Fifteen patients received p28 intravenously under an accelerated titration 3 + 3 dose escalation design. p28 was well tolerated with no significant adverse events, suggesting that it appeared to have antitumor activity in patients with advanced tumors (106). Another Phase I trial of p28 as a single agent in children with central nervous system (CNS) tumors was conducted. Children with recurrent or progressive CNS tumors received p28 intravenously at a dose of 4.16 mg/kg/dose (the recommended Phase II dose for adults) using a rolling 6 study design. While adult p28 doses were tolerated in adolescents, similar results were observed, further suggesting that p28-based treatments could be administered in all age groups. Results from these trials established that p28 was safe and well tolerated at the recommended Phase II dose (RP2D). Although p28 shows preliminary efficacy, further development of the drug in combination with other agents may prove more promising (110).

### 4.3 The clinical trial of ST101

ST101 is a leucine zipper peptide with the ability to penetrate cells, and it is expected to be used for clinical treatment of cancer (106). ST101 is currently undergoing clinical trials for brain cancer and other solid tumors. In particular, ST101 has shown impressive anti-tumor activity in subcutaneous xenograft models. According to a report by ClinicalTrials.gov in July 2020, a “ Phase I-II study of ST101 in advanced solid tumor patients” (NCT04478279) was conducted. The abstract presented for the first time at the November 2022 meeting of the Society for Neuro-Oncology reported the results of a Phase II study of the first class peptide antagonist ST101 in recurrent glioblastoma. This study recruited adult cancer patients who relapsed after a standard treatment regimen. The treatment with ST101 involves intravenous injections of 500 mg per week. After 18 weeks of observation, only 1 out of 7 patients showed a partial response according to mRANO criteria. Although the study is still in its early phase, the apparent safety and efficacy of the drug are currently encouraging (111, 112).

## 5 Conclusions

Over the past decade, numerous studies have elucidated that cell-penetrating peptides (CPPs), acting as carriers for therapeutic agents, hold significant promise in the treatment of various cancers by efficiently delivering multiple biologically active cargos into cells, particularly in the context of tumor therapy. CPPs not only exhibit low cytotoxicity and high transduction efficiency but also facilitate the selective delivery of anticancer drugs, thereby reducing toxic effects on normal tissues. Although CPPs have extensive application potential in both fundamental research and clinical trials, CPPs still face challenges such as insufficient biochemical stability, short half-life, and the tendency to form cleaved peptides upon modification with drug molecules. Consequently, covalently coupling CPPs with biomolecules to form stable chemical bonds, ensuring the integrity of both the CPP and cargo, or non-covalently assembling of CPPs with nanocarriers, liposomes or micelles to significantly enhance delivery efficiency, while utilizing non-natural amino acids to improve pharmacokinetic properties, has become a focus of future research.
